# Development and psychometric validation of a community microbiology literacy scale in the context of infectious diseases, antimicrobial resistance, and vaccination

**DOI:** 10.1093/femsle/fnag107

**Published:** 2026-09-17

**Authors:** Rukiye Aslan, Ezgi Ağadayı, Aysel Arslan, Sait Bardakçı

**Affiliations:** Faculty of Pharmacy, Department of Pharmaceutical Microbiology, Sivas Cumhuriyet University, Sivas, 58140, Türkiye; Faculty of Medicine, Department of Medical Education, Sivas Cumhuriyet University, Sivas, 58140, Türkiye; Faculty of Education, Department of Educational Sciences, Sivas Cumhuriyet University, Sivas, 58140, Türkiye; Faculty of Economics and Administrative Sciences, Department of Quantitative Methods, Sivas Cumhuriyet University, Sivas, 58140, Türkiye

**Keywords:** microbiology literacy, antimicrobial resistance, infection prevention, public health microbiology, scale validation

## Abstract

Microbiology literacy has become increasingly important for public health and societal resilience, particularly in the context of emerging infectious diseases, antimicrobial resistance, and misinformation. However, standardized instruments for assessing Microbiology literacy at the community level remain limited. This study aimed to develop a valid and reliable tool to assess Microbiology literacy in the general population and to examine its multidimensional structure. This methodological, cross-sectional study developed the Community Microbiology Literacy Scale (CMLS). An initial item pool was generated based on international Microbiology books and World Health Organization (WHO) guidelines, followed by expert evaluation and content validity assessment using the Davis technique. Data were collected from 835 adults aged ≥18 years. Exploratory factor analysis (EFA) was conducted using data from 498 participants without imposing an a priori dimensional structure. A conceptually coherent four-factor structure emerged from the data, and the factors were named according to the content of their constituent items. This structure was subsequently tested by confirmatory factor analysis (CFA) in an independent subsample of 337 participants. The EFA identified a four-factor structure comprising Basic Microbiology Knowledge, Infection Control Awareness, Antibiotic and Antimicrobial Resistance Awareness, and Vaccine Awareness, explaining 60.66% of the total variance. CFA confirmed acceptable model fit. The scale demonstrated high internal consistency [Cronbach’s alpha (α) = 0.925], with subscale Cronbach’s α values ranging from 0.793 to 0.896. Higher Microbiology literacy scores were associated with older age, higher educational attainment, being a healthcare professional, prior Biology or Microbiology education, and awareness of the term “Microbiology” (*P* < .05). No significant differences were observed according to place of residence. The CMLS is a valid, reliable, and multidimensional instrument for assessing community-level Microbiology literacy and may support targeted educational and public health interventions.

## Teaser

This study introduces and validates the first comprehensive scale for assessing community microbiology literacy, supporting public health education and awareness of infections, antimicrobial resistance, and vaccination.

## Introduction

Microbiology is a fundamental scientific discipline that influences nearly every aspect of life, ranging from human health and environmental sustainability to food safety and biotechnological innovation. It encompasses the study of the structure, functions, transmission pathways, and interactions of microorganisms and highlights their dual role, not only as potential pathogens but also as essential contributors to ecosystem balance, nutrient cycling, and human well-being (Sánchez-Angulo et al. 2021, Timmis et al. 2022, Bradshaw 2021, Davies and Davies 2010, Fidiastuti et al. 2024).

In this context, individuals’ levels of Microbiology-related knowledge play a decisive role not only in shaping health-related behaviors but also in protecting public health. The COVID-19 pandemic clearly demonstrated the consequences of insufficient Microbiological understanding at both individual and societal levels. Limited knowledge regarding transmission routes, infection control, immune responses, and vaccination was associated with ineffective health behaviors and increased disease spread. Likewise, inadequate awareness of issues such as antibiotic misuse and vaccine protection has contributed to the growing burden on healthcare systems. Together these observations suggest that insufficient Microbiology-related knowledge may contribute to nonevidence-based decision-making with significant public health implications. Consequently, Microbiology literacy has emerged as a key determinant of informed decision-making (Allgaier et al. 2013, Funk and Rainie 2015).

Educational approaches based on active, inquiry-oriented, and problem-based learning have been shown to enhance students’ scientific literacy by strengthening evidence evaluation, critical thinking, and problem-solving skills (Merkel 2016, Davis et al. 2020). Similarly, Microbiology-centered citizen science initiatives promote public engagement and experiential learning, increasing awareness of the role of microorganisms in health and the environment (Timmis et al. 2020, Timmis 2023). However, these approaches primarily evaluate learning outcomes or participant engagement rather than providing standardized instruments for assessing Microbiology literacy as a multidimensional construct at the community level.

Several assessment tools have been developed to evaluate learning in Microbiology education. Among the most widely used assessment tools are concept inventories, particularly the Microbiology Concept Inventory (MCI) and Microbiology for Health Sciences Concept Inventory (MHSCI). The MCI, developed for undergraduate Microbiology education, evaluates students’ conceptual understanding across the core concepts defined by the American Society for Microbiology Curriculum Guidelines (Paustian et al. 2017, Rennpferd et al. 2023). Similarly, the MHSCI was specifically designed for health sciences students to assess learning outcomes in health-related Microbiology courses (Seitz et al. 2017). Although these instruments provide standardized measures of conceptual learning, they were developed primarily for educational settings and are not designed to assess Microbiology literacy as a multidimensional construct applicable to the general population.

Scientific literacy encompasses the ability to use evidence-based reasoning in decision-making and to engage critically with science-related societal issues. Although Microbiology provides an important context for developing these competencies, Microbiology literacy itself has not yet been sufficiently conceptualized or operationalized as a distinct, measurable, and multidimensional construct (Laugksch 2000, Norris and Phillips 2003, Savage and Jude 2014, Timmis et al. 2020, Timmis 2023).

Existing empirical studies in Microbiology-related fields have primarily focused on specific domains, such as microbiota awareness, hygiene practices, or antimicrobial use, and have often been conducted in restricted and relatively homogeneous populations, particularly students (Mustofa et al. 2025, Amorim et al. 2025). Although these studies have provided valuable insights into Microbiology-related knowledge and awareness, their scope remains limited for assessing Microbiology literacy as a comprehensive, multidimensional construct at the community level. In addition, recent international initiatives have emphasized the need for broader, societally relevant frameworks that promote Microbiology literacy beyond traditional educational settings (Timmis et al. 2024).

Recent studies have demonstrated that Microbiology literacy should be addressed not only in terms of cognitive knowledge but also by incorporating attitudinal and behavioral dimensions (Timmis et al. 2019). Individuals with adequate Microbiological knowledge have been shown to adopt more appropriate hygiene and infection control practices and to demonstrate greater awareness of vaccination and antimicrobial resistance (Bloomfield et al. 2007). In this respect, Microbiology literacy has been recognized as an important contributor to evidence-based decision-making at individual, societal, and policy levels by promoting public health awareness and reducing the risk of misinformation-driven behaviors. Notably, the International Microbiology Literacy Initiative (IMiLI) advocates integrating Microbiology education into curricula from the early stages of education to strengthen Microbiology literacy across society (Timmis et al. 2020).

As a result, Microbiology literacy remains insufficiently conceptualized, and no standardized, multidimensional instrument is currently available to assess community-level Microbiology literacy particularly in countries where such instruments have not yet been developed, including Türkiye. To address this gap, the present study adopted a community-based design with a large and diverse sample to develop and validate a multidimensional measure of Microbiology literacy at the population level. Accordingly, this study aimed to develop and psychometrically validate the Community Microbiology Literacy Scale (CMLS), explore its underlying dimensional structure, and examine differences in Microbiology literacy across sociodemographic characteristics.

## Materials and methods

### Study design and setting

This study was designed as a methodological, cross-sectional scale development study. The primary aim was to develop a valid and reliable instrument to assess Microbiology literacy among adults in the community. Data were collected between 1 May and 1 August 2025, and psychometric analyses were conducted to evaluate the validity and reliability of the scale.

The scale development process included item generation, content validity assessment, exploratory factor analysis (EFA), confirmatory factor analysis (CFA), and reliability analyses. To ensure robust construct validity, EFA and CFA were performed on independent data sets.

Ethical approval for the study was obtained from the Sivas Cumhuriyet University (Türkiye), Health Sciences Research Ethics Committee (approval date: 24.04.2025; approval number: 2025–04/146).

### Scale development process

During the item pool development process, CMLS was conceptualized within the dimensions of Basic Microbiology Knowledge, Hygiene and Infection Control, Antibiotic and Vaccine Awareness, and the interaction between Microbiology and daily life. Item generation was based on fundamental Microbiology books, World Health Organization (WHO) guidelines on infection prevention and control, antimicrobial resistance, vaccination, food safety, and probiotics, as well as relevant literature on Microbiology education and public knowledge (WHO 2009, 2014, 2020, 2024, Timmis et al. 2019, Murray et al. 2025).

Guided by this theoretical framework and literature findings, an item pool was generated to assess Microbiology literacy in the general population. Care was taken to ensure that the items were clear, understandable, and designed to measure a single knowledge or behavior domain. The items were structured using a 5-point Likert-type response format (1 = strongly disagree, 2 = disagree, 3 = neither agree nor disagree, 4 = agree, 5 = strongly agree).

Following the item pool development process, the draft version of the scale consisted of 103 items. To evaluate the scope and content representativeness of the Microbiology literacy construct, the draft scale was reviewed by eight subject-matter experts, who assessed each item in terms of relevance, clarity, and representativeness. Item-level Content Validity Index (I-CVI) values were calculated using the Davis technique (Davis 1992). In accordance with the recommendations of Polit and Beck, items with an I-CVI value of 0.78 or higher were considered acceptable for expert panels consisting of six to ten experts and were retained in the scale (Polit and Beck 2006) (Table S1). Following the content validity assessment, 25 items met the predefined inclusion criterion and were retained in the draft version of the scale, whereas 78 items with insufficient content validity were excluded. The development of the CMLS, including the item selection and content validation process, is presented in Fig. S1.

### Participants and sampling

The target population of the study consisted of the general adult population. Accordingly, individuals aged 18 years and older living in Sivas; Türkiye, who were literate and able to use a smartphone, were considered eligible to participate in the study. Eligibility was determined through self-reported screening questions administered at the beginning of the online questionnaire, and only participants meeting all predefined inclusion criteria were allowed to proceed with the survey.

Participants were reached through convenience sampling using easily accessible channels, including social media platforms, email groups, announcements disseminated through universities and public institutions, and the researchers’ professional and social networks.

In scale development studies, it is recommended that the sample size for factor analysis should be at least 5–10 times the number of items included in the scale. Moreover, conducting EFA and CFA on independent data sets is advised to ensure robust construct validity. Based on these recommendations and considering that the draft version of the scale consisted of 25 items, the minimum required sample size was calculated as 25 × 10 = 250 participants for each analysis. Accordingly, a total sample size of at least 500 participants was targeted to perform EFA and CFA on separate samples.

Prior to the main data collection, a pilot study was conducted with 30 participants to evaluate the clarity, comprehensibility, and applicability of the scale items. No incomprehensible or ambiguous items were identified during the pilot testing, and therefore no revisions were made to the scale before proceeding to the main study.

### Psychometric evaluation

Construct validity was examined using EFA. Prior to factor extraction, sampling adequacy and factorability were assessed using the Kaiser–Meyer–Olkin (KMO) measure and Bartlett’s test of sphericity. EFA was conducted using the Principal Axis Factoring method with Varimax rotation. Factors with eigenvalues greater than 1 were retained according to Kaiser’s criterion (Kaiser 1960). Factor loadings of ≥0.30 were considered acceptable, as recommended in psychometric and multivariate analysis literature (Tabachnick and Fidell 2013, Hair et al. 2018). Items that did not load meaningfully on any factor or demonstrated cross-loadings were removed from the scale. The number of factors, factor loadings, communalities, and explained variance were evaluated to determine the underlying factor structure.

CFA was performed on an independent subsample to verify the factor structure obtained from the EFA. Model fit was evaluated using the chi-square/degrees of freedom ratio (CMIN/DF), Goodness-of-Fit Index (GFI), Adjusted Goodness-of-Fit Index (AGFI), Comparative Fit Index (CFI), Incremental Fit Index (IFI), Tucker–Lewis Index (TLI), and Root Mean Square Error of Approximation (RMSEA). Based on recommended criteria in the literature, a CMIN/DF value below 5 and GFI, AGFI, CFI, IFI, and TLI values of 0.90 or higher were considered indicative of acceptable model fit (Hu and Bentler 1999, Marsh et al. 2004, Brown 2015, Byrne 2016, Kline 2023). RMSEA values <0.05 were considered indicate good fit, values between 0.05 and 0.08 of reasonable fit, values between 0.08 and 0.10 of mediocre but acceptable fit, and values >0.10 of poor fit (Browne and Cudeck 1993, Byrne 2016). Modification indices (MIs) were examined, and error covariances were added only between items demonstrating clear semantic overlap and supported by theoretical justification. Such model modifications are recommended only when they are theoretically meaningful and are considered an acceptable practice in CFA studies (Brown 2015, Byrne 2016, Kline 2023).

Internal consistency reliability of the scale was assessed using Cronbach’s α coefficient. Cronbach’s α values were calculated for both the overall scale and each subscale. A Cronbach’s α coefficient of 0.70 or higher was considered indicative of acceptable internal consistency reliability (Tabachnick and Fidell 2013, Hair et al. 2018). Item–total correlation coefficients were examined to evaluate the contribution of individual items to the overall reliability, and values of 0.30 or higher were considered satisfactory. Items with item–total correlation values below 0.30 were considered for removal. Additionally, Cronbach’s α values if item deleted were examined to determine whether the removal of any item would improve internal consistency.

### Statistical analysis

Statistical analyses were performed using IBM SPSS Statistics (version 23.0) and AMOS Graphics. Sociodemographic characteristics were obtained through self-reported responses collected as part of the online questionnaire. Descriptive statistics were calculated for sociodemographic characteristics and scale scores and are presented as frequencies, percentages, means, and standard deviations, as appropriate.

The total sample (*N* = 835) was randomly divided into two independent subsamples. The first subsample (*n* = 498) was used for EFA, whereas the second subsample (*n* = 337) was used for CFA to cross-validate the factor structure. Content validity analyses, EFA, reliability analyses, independent samples *t*-tests, one-way analysis of variance (ANOVA), and Tukey *post hoc* tests were conducted using IBM SPSS Statistics. CFA and model fit analyses were performed using AMOS Graphics. Content validity was assessed using the CVI based on expert ratings according to the Davis technique. Construct validity and reliability analyses were conducted as described in the Psychometric Evaluation section. For sociodemographic comparisons, categories with small sample sizes were combined on a conceptually meaningful basis. The 46–55 and ≥56-year age groups were combined as ≥46 years, primary- and middle-school education categories as below high school, and television/radio and newspapers/magazines as traditional media. The original categories were retained in Table 1 for descriptive purposes, whereas the combined categories were used in the comparative analyses presented in Table 5. Differences in CMLS scores according to sociodemographic and educational characteristics were analysed using independent-samples *t*-tests or one-way ANOVA, as appropriate. Homogeneity of variances was assessed using Levene’s test. Tukey’s *post hoc* test was used when the ANOVA results were statistically significant. A *P-*value of <.05 was considered statistically significant.

**Table 1:** Sociodemographic characteristics of the participants (*N* = 835).

Variable	Group	*n*	%
Age (years)	18–25	569	68.1
	26–35	112	13.4
	36–45	102	12.2
	46–55	37	4.4
	≥56	15	1.9
Education level	Primary school	16	1.9
	Middle school	19	2.3
	High school	241	28.9
	Undergraduate (Bachelor’s)	488	58.4
	Graduate (Master’s/PhD)	71	8.5
Occupation	Healthcare sector	91	10.9
	Education sector	101	12.1
	Public service	47	5.6
	Private sector	97	11.6
	Student	499	59.8
Place of residence	Village	100	12.0
	District	100	12.0
	City	502	60.1
	Metropolitan city	133	15.9
Primary source of health-related information	Social environment and friends	78	9.3
	Internet and social media	475	56.9
	Traditional media	19	2.3
	Healthcare professionals	263	31.5
Previous education in biology	Yes	363	43.5
	No	472	56.5
Previous education in microbiology	Yes	176	21.1
	No	659	78.9
Awareness of the meaning of “Microbiology”	Yes	573	68.6
	No	262	31.4
Total		835	100.0

*Note*. For the comparative analyses presented in Table 5, categories with small sample sizes were combined into broader categories.

## Results

### Demographic characteristics of the participants

A total of 835 adults participated in the study. Most participants were aged 18–25 years (*n* = 569; 68.1%). More than half of the participants had a university-level education (*n* = 488; 58.4%). Students constituted the largest occupational group (*n* = 499; 59.8%). Regarding access to health-related information, 56.9% (*n* = 475) of the participants reported frequently obtaining such information through the internet and social media. Additionally, 56.5% (*n* = 472) stated that they had never received formal education in Biology, while 78.9% (*n* = 659) reported never having received any education in Microbiology. Furthermore, 68.6% (*n* = 573) of the participants indicated that they understood the meaning of the term Microbiology. The sociodemographic characteristics of the participants are presented in Table 1.

### Item pool development and content validity

Content validity assessment of the initial 103-item pool resulted in the retention of 25 items and the exclusion of 78 items that did not meet the predefined content validity criterion. The I-CVI values of the retained items ranged from 0.875 to 1.000. The Scale-Level Content Validity Index based on the average method (S-CVI/Ave) was 0.995. Detailed item-level CVI results are presented in Table S2.

### Construct validity

The KMO value was 0.931, indicating excellent sampling adequacy (Tabachnick and Fidell 2013, Hair et al. 2018). Bartlett’s test of sphericity was significant [χ² (55) = 7020.759, *P* < .001)], confirming that the correlation matrix was suitable for factor analysis. EFA using the Principal Axis Factoring method with Varimax rotation extracted four factors with eigenvalues greater than 1, accounting for 60.66% of the total variance (Factor 1: 40.38%, Factor 2: 9.49%, Factor 3: 6.05%, and Factor 4: 4.72%). Communalities ranged from 0.402 to 0.791, and standardized factor loadings for the retained items ranged from 0.308 to 0.854 (see Table 2). Based on the conceptual content of the retained items, the four factors were labeled as Basic Microbiology Knowledge, Infection Control Awareness, Antibiotic and Antimicrobial Resistance Awareness, and Vaccine Awareness. Items 21, 23, and 24 were excluded from the scale because they did not demonstrate meaningful loadings on any factor, exhibiting factor loadings below the predefined threshold of 0.30 across all factors.

**Table 2:** EFA results of the CMLS.

Item code	Factor 1 basic microbiology knowledge	Factor 2 infection control awareness	Factor 3 antibiotic and antimicrobial resistance awareness	Factor 4 vaccine awareness	Communality
Item 1	0.703				0.559
Item 2	0.604		0.382		0.557
Item 3	0.736	0.374	0.310		0.653
Item 4	0.713				0.641
Item 5	0.573		0.308		0.563
Item 6	0.721			0.416	0.703
Item 7	0.729			0.371	0.690
Item 8	0.561				0.439
Item 9		0.757			0.720
Item 10		0.854			0.791
Item 11		0.748			0.632
Item 12		0.807			0.762
Item 13	0.328	0.534		0.311	0.544
Item 14			0.483		0.425
Item 15			0.530		0.402
Item 16			0.663		0.620
Item 17		0.329	0.707		0.647
Item 18				0.672	0.668
Item 19			0.400	0.645	0.662
Item 20				0.770	0.661
Item 21					0.555
Item 22		0.508	0.445		0.572
Item 23					0.540
Item 24					0.578
Item 25		0.467	0.579		0.584

### CFA

The total sample (*N* = 835) was randomly divided into two independent subsamples for EFA and CFA. Approximately 60% of participants were allocated to the EFA subsample (*n* = 498) and 40% to the CFA subsample (*n* = 337). A larger subsample was assigned to EFA, because EFA generally requires a larger sample size to obtain stable factor solutions and reliable factor loading estimates. The EFA subsample exceeded commonly recommended participant-to-item ratios (at least 10 participants per item) and met criteria for a “very good” sample size according to Comrey and Lee (1992). The CFA subsample also exceeded the commonly recommended minimum sample size of 200 participants for CFA and structural equation modeling (Kline 2023), providing adequate statistical power for model validation. After inspecting the MIs, five theoretically justifiable error covariances were added between items with overlapping semantic content. No items were removed from the model. This respecification substantially improved overall model fit. Model fit statistics are presented in Table 3. The final CFA model of the CMLS is shown in Fig. 1.

**Figure 1:**
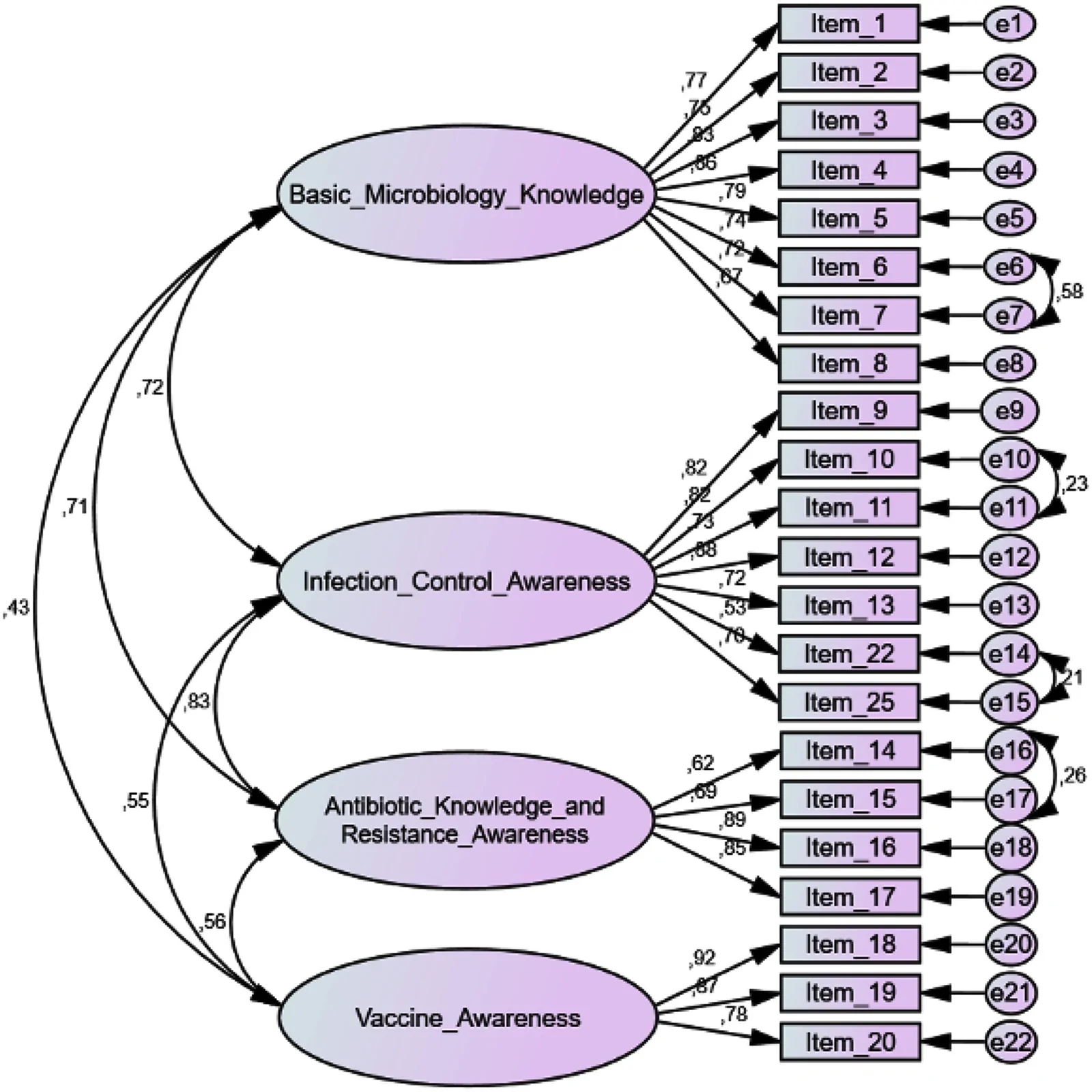
CFA model of the CMLS. Ellipses represent the latent factors, rectangles represent the observed items, and circles (e1–e22) indicate measurement error terms. Single-headed arrows represent standardized factor loadings, double-headed curved arrows represent correlations between latent factors or correlated error terms, and the numerical values correspond to standardized estimates obtained from the CFA. Path diagram of the four-factor Community Microbiology Literacy Scale showing four latent dimensions, 22 observed items, standardized factor loadings, correlations among latent factors, and selected correlated error terms.

**Table 3:** Model fit indices for the four-factor CMLS’s CFA model.

Fit index	Value
CMIN/DF	3.653
GFI	0.831
AGFI	0.785
CFI	0.902
IFI	0.903
TLI	0.886
RMSEA	0.089

### Internal consistency reliability

The internal consistency reliability of the scale was assessed using Cronbach’s α. The overall Cronbach’s α coefficient was α = 0.925. Item–total correlations ranged between 0.438 and 0.680, and Cronbach’s α if item deleted values were between 0.920 and 0.925, suggesting that no item substantially reduced the reliability of the scale (see Table 4).

**Table 4:** Internal consistency analysis results for the Turkish version of the CMLS (EFA sample, *N* = 498).

Item code	Item-total correlation	Cronbach’s α if item deleted
Item 1	0.483	0.923
Item 2	0.522	0.923
Item 3	0.549	0.922
Item 4	0.601	0.921
Item 5	0.631	0.921
Item 6	0.558	0.922
Item 7	0.462	0.924
Item 8	0.514	0.923
Item 9	0.680	0.920
Item 10	0.635	0.921
Item 11	0.569	0.922
Item 12	0.660	0.920
Item 13	0.656	0.920
Item 14	0.569	0.922
Item 15	0.497	0.923
Item 16	0.630	0.921
Item 17	0.630	0.921
Item 18	0.557	0.922
Item 19	0.598	0.921
Item 20	0.438	0.925
Item 22	0.676	0.920
Item 25	0.624	0.921
Cronbach’s α of scale and subscales		
CMLS	0.925	
Factor 1	0.870	
Factor 2	0.896	
Factor 3	0.793	
Factor 4	0.833	

### Participants’ community microbiology literacy

The mean total CMLS score of the participants was 3.82 ± 0.62. The mean subscale scores were 3.63 ± 0.77 for the Basic Microbiology Knowledge subscale, 4.06 ± 0.69 for the Infection Control Awareness subscale, 3.85 ± 0.77 for the Antibiotic and Antimicrobial Resistance Awareness subscale, and 3.75 ± 0.89 for the Vaccine Awareness subscale. Total CMLS scores differed significantly according to age (*P* = .013), education level (*P* < .001), occupation (*P* < .001), primary source of health-related information (*P* = .015), previous education in Biology (*P* < .001) and Microbiology (*P* < .001), and awareness of the meaning of the term “Microbiology” (*P* < .001). Tukey’s *post hoc* tests showed that participants aged 18–25 years had significantly lower scores than all other age groups. Participants with postgraduate education had significantly higher scores than those in all other education groups, while participants working in the healthcare sector had significantly higher scores than those in all other occupational groups. Participants whose primary source of health-related information was their social circle and friends had significantly lower scores than those who obtained information from traditional media or healthcare professionals. No significant difference was observed according to place of residence (*P* = .670) (Table 5).

**Table 5:** Differences in CMLS scores according to sociodemographic and educational characteristics.

Age	Mean	SD	*P*
18–25	3.7	0.6	**.013**
26–35	3.9	0.5	
36–45	3.9	0.5	
>46	3.9	0.6	
Education level			
Below high school	3.7	0.7	**<.001**
High school	3.7	0.6	
Undergraduate	3.8	0.6	
Graduate	4.1	0.5	
Occupation			
Healthcare sector	4.1	0.6	**<.001**
Education sector	3.8	0.5	
Public service	3.8	0.5	
Private sector	3.8	0.6	
Student	3.7	0.6	
Place of residence			
Village	3.8	0.5	.670
District	3.9	0.5	
City	3.8	0.6	
Metropolitan city	3.8	0.7	
Primary source of health-related information			
Social environment & friends	3.6	0.6	**.015**
Internet & social media	3.8	0.6	
Traditional media	4.0	0.4	
Healthcare professionals	3.9	0.6	
Previous education in Biology			
Yes	4.0	0.6	**<.001**
No	3.7	0.6	
Previous education in microbiology			
Yes	4.2	0.6	**<.001**
No	3.7	0.5	
Awareness of the meaning of “Microbiology”			
Yes	4.0	0.6	**<.001**
No	3.5	0.6	

*Note*. For comparative analyses, the 46–55 and ≥56-year age groups were combined as ≥46 years, primary**-** and middle**-**school education categories as below high school, and television/radio and newspapers/magazines as traditional media. Bold values indicate statistically significant differences (*P* < 0.05).

### Final version of the CMLS

Following expert evaluation, item refinement, EFA, and CFA, the final version of CMLS was established. The final scale consisted of 22 items organized into four dimensions: Basic Microbiology Knowledge (8 items), Infection Control Awareness (7 items), Antibiotic and Resistance Awareness (4 items), and Vaccine Awareness (3 items). All items were retained based on conceptual relevance and satisfactory psychometric performance during the validation process. Responses were scored using a five-point Likert scale, ranging from 1 (strongly disagree) to 5 (strongly agree), with higher scores indicating greater levels of community Microbiology literacy. The final English version of the scale is presented in Table 6, while the original Turkish version is provided in Table S3.

**Table 6:**
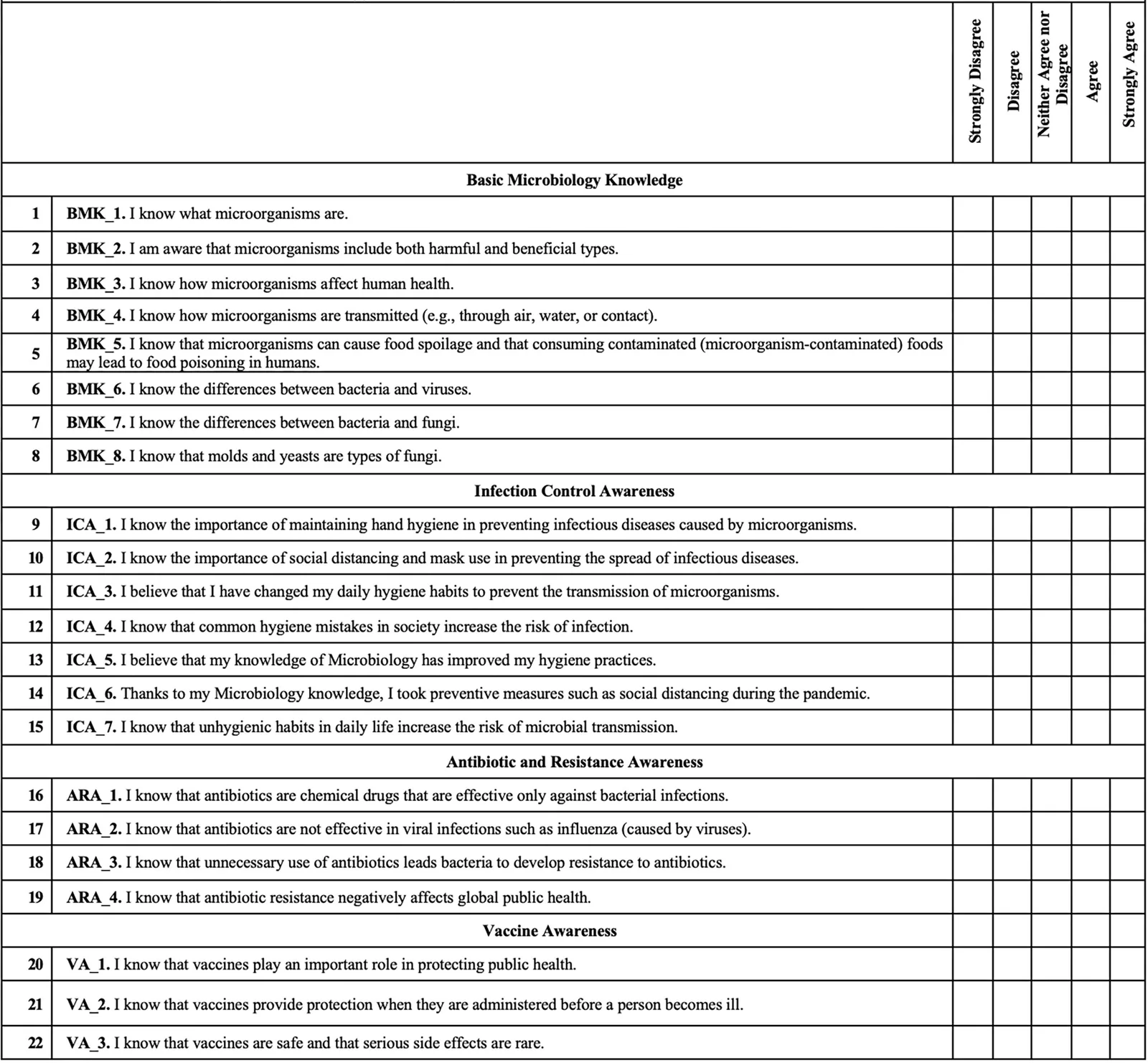
Commmunity Microbiology Literacy Scale.

*Note*. The final version of CMLS consisted of 22 items organized into four dimensions; BMK: Basic Microbiology Knowledge; ICA: Infection Control Awareness; ARA: Antibiotic and Resistance Awareness; VA: Vaccine Awareness.

## Discussion

This study developed and psychometrically validated the CMLS, a multidimensional instrument designed to assess Microbiology literacy at the community level. The findings demonstrated satisfactory psychometric performance, including content validity, construct validity, and internal consistency, supporting the use of the CMLS as a reliable tool for community-based assessment of Microbiology literacy.

The findings suggest that Microbiology literacy is not limited to the acquisition of scientific knowledge but also involves the ability to translate this knowledge into health-related awareness and informed behaviors. The identified dimensions collectively reflect key competencies required for understanding microorganisms, adopting infection prevention practices, promoting appropriate antimicrobial use, and supporting evidence-based vaccination decisions. This conceptual framework is consistent with recent perspectives emphasizing that Microbiology literacy should integrate scientific understanding with behaviors that contribute to individual and community health (Timmis et al. 2019, 2020).

Content validation demonstrated strong expert agreement regarding the relevance and clarify of retained items. The item-level I-CVI validity values ranged from 0.875 to 1.00, indicating strong expert agreement regarding the relevance and clarity of the retained items. Likewise, the high S-CVI/Ave (0.995) indicates that the scale adequately represents the intended domains of Microbiology literacy. These findings are consistent with previous recommendations indicating that high CVIs strengthen confidence in the representativeness and conceptual coherence of newly developed measurement instruments (Davis 1992, Polit and Beck 2006).

The construct validity findings further supported the proposed structure of the CMLS. The high KMO value (0.931) and significant Bartlett’s test confirmed that the data were suitable for factor analysis. The EFA identified a four-factor structure explaining 60.66% of the total variance. This level of explained variance is considered acceptable for multidimensional constructs in social and behavioral sciences (Costello and Osborne 2005) and provides further evidence that Microbiology literacy comprises multiple interrelated domains rather than a single knowledge-based construct.

The observed factor loadings (0.308–0.854) and communalities (0.402–0.791) indicated that the retained items were appropriately associated with their respective dimensions and contributed to the internal coherence of the scale. These findings are consistent with methodological recommendations indicating that adequate factor loadings and communalities provide evidence that retained items appropriately represent the underlying construct (Costello and Osborne 2005). Collectively, these findings support the structural coherence of the CMLS and demonstrate its suitability for assessing multiple components of Microbiology literacy in community settings.

CFA further supported the proposed four-factor model. To improve model fit, a limited number of correlated error terms were introduced only between items with overlapping semantic content when supported by MIs and theoretical justification. Such theoretically justified covariance modifications are considered acceptable in CFA when applied cautiously and interpreted within the conceptual framework of the instrument (Brown 2015, Byrne 2016). These modifications improved model fit while preserving the theoretical structure of the scale.

The introduction of a limited number of theoretically justified correlated error terms improved model fit without altering the underlying factor structure of the CMLS. Such modifications are considered acceptable in CFA when they reflect similarities in item wording or overlapping behavioral contexts rather than model misspecification (Marsh et al. 2004, Brown 2015, Byrne 2016, Kline 2023). In the present study, correlated error terms were introduced only within factors, minimizing the risk of overfitting while preserving the theoretical integrity of the measurement model. Although the TLI value was slightly below the recommended threshold and the RMSEA value marginally exceeded the conventional criterion, the remaining fit indices supported an acceptable overall model fit, providing additional evidence for the structural validity of the CMLS (Hu and Bentler 1999, Marsh et al. 2004).

Among the four dimensions, Infection Control Awareness showed the highest mean score, whereas Basic Microbiology Knowledge had the lowest. This pattern may suggest that public awareness of infection prevention practices has increased following the COVID-19 pandemic, whereas fundamental understanding of Microbiological concepts has not necessarily improved to the same extent. Previous studies have similarly shown that individuals often adopt recommended preventive behaviors when exposed to public health campaigns and health information, even though broader scientific understanding may remain limited (van der Linden et al. 2020, Wang et al. 2021, Aslan et al. 2023, Ceylan and Koç 2024). These findings highlight the importance of strengthening educational strategies that improve not only compliance with preventive measures but also fundamental Microbiological understanding, thereby supporting informed health-related decision-making.

The findings address an important gap in the literature by providing a standardized instrument for the comprehensive assessment of Microbiology literacy at the community level. Although previous instruments have evaluated related constructs such as health literacy, e-health literacy, antibiotic knowledge, vaccination attitudes, or infection control practices, they have generally focused on individual domains rather than Microbiology literacy as a multidimensional construct (Martin and Petrie 2017, Aslan et al. 2023, Zanini et al. 2023). By integrating multiple complementary domains within a single validated framework, the CMLS enables a more comprehensive assessment of Microbiology literacy than previously available instruments and provides a foundation for future comparative research across different populations and settings (Timmis et al. 2019, 2022, Bradshaw 2021).

Beyond its psychometric performance, the present study demonstrated significant associations between Microbiology literacy and several sociodemographic characteristics, providing additional insight into factors associated with Microbiology literacy in the community. Older participants achieved slightly higher Microbiology literacy scores than younger participants, which may reflect cumulative educational experiences, repeated exposure to health-related information, and greater familiarity with infectious diseases acquired over time. Similar associations between age and health literacy have been reported previously, although the magnitude of this relationship appears to vary across different populations and sociocultural contexts (Sørensen et al. 2012, van der Linden et al. 2020). Educational attainment also emerged as an important determinant of Microbiology literacy, with participants holding graduate degrees demonstrating the highest literacy scores. This finding is consistent with previous evidence indicating that higher educational attainment enhances individuals’ ability to acquire, critically evaluate, and apply scientific and health-related information (Nutbeam 2008, Sørensen et al. 2012). Likewise, healthcare professionals, as well as participants with previous education in Biology or Microbiology, achieved significantly higher literacy scores than other participants. This finding is consistent with previous studies demonstrating that formal Microbiology education strengthens Microbiology literacy and promotes a broader understanding of the role of microorganisms in health and disease (Timmis 2023, Fidiastuti et al. 2024). Given their formal education and routine exposure to Microbiological concepts, infection prevention, and antimicrobial stewardship, these findings are expected and further support the ability of the CMLS to distinguish between groups with different levels of Microbiology-related knowledge and experience. Furthermore, participants who reported being familiar with the term “Microbiology” also demonstrated significantly higher literacy scores, suggesting that familiarity with basic Microbiological concepts may reflect broader Microbiology literacy rather than simple recognition of terminology. Collectively, these findings indicate that Microbiology literacy is closely associated with educational exposure and disciplinary experience, supporting the educational framework proposed by the IMiLI and recent recommendations advocating societally relevant Microbiology education (Timmis et al. 2019, 2022, 2024).

Another noteworthy finding was the significant association between Microbiology literacy and participants’ primary sources of health-related information. Participants who reported healthcare professionals as their primary source of health-related information demonstrated significantly higher Microbiology literacy scores than those relying mainly on family members or friends. This finding is consistent with previous evidence indicating that information obtained from trusted and professionally curated sources is associated with higher levels of health literacy, greater confidence in scientific information, and more informed health-related decision-making (Nutbeam 2008, Sørensen et al. 2012, Chen et al. 2018, Riiser et al. 2020, Rennpferd et al. 2023). Although participants who reported traditional media as their primary information source also showed relatively high literacy scores, this observation should be interpreted cautiously because of the small subgroup size. In contrast, no significant association was observed between Microbiology literacy and place of residence, suggesting that educational opportunities and access to reliable health information may have a greater influence on Microbiology literacy than geographical location. From a public health perspective, these findings emphasize that improving Microbiology literacy requires not only expanding educational opportunities but also strengthening access to accurate, evidence-based information delivered through reliable communication channels.

The potential applications of the CMLS extend beyond psychometric validation. The scale may serve as a practical tool for monitoring Microbiology literacy across different population groups, evaluating the effectiveness of educational and public health interventions, and identifying communities that may benefit from targeted Microbiology education. Furthermore, its application in diverse populations will facilitate future cross-cultural validation and support international comparisons of Microbiology literacy using a standardized measurement approach (Timmis et al. 2019, 2022).

Despite its strengths, this study has several limitations. Data were collected from a single geographical region and relied on self-reported responses, which may limit the generalizability of the findings. Although the overall sample size was adequate for psychometric validation, some sociodemographic subgroups remained unequal in size even after conceptually appropriate categories were combined; therefore, the related comparisons should be interpreted with caution. In addition, the cross-sectional design precludes causal inferences regarding the observed relationships. Future studies should focus on evaluating the cross-cultural validity, measurement invariance, and responsiveness of the CMLS across more diverse populations and settings. Longitudinal research is also needed to examine changes in Microbiology literacy over time and to determine the effectiveness of educational and public health interventions aimed at improving Microbiology literacy.

In conclusion, the CMLS is a valid and reliable instrument for assessing Microbiology literacy in the general population. By providing a standardized and multidimensional measure, it addresses an important gap in the current literature and provides a practical tool for research, educational evaluation, and public health practice. Its application across diverse populations may help identify Microbiology literacy needs, inform the development of evidence-based educational and public health interventions, and support future national and international research on Microbiology literacy.

## Supplementary Material

fnag107_Supplemental_Files

## Data Availability

The datasets generated during and/or analysed during the current study are available from the corresponding author on reasonable request.
