# Supplementary material for: Development and psychometric validation of a community microbiology literacy scale in the context of infectious diseases, antimicrobial resistance, and vaccination
Source: FEMS Microbiol Lett. 2026 Sep 17;373:fnag107. doi: 10.1093/femsle/fnag107 (PMC13618252; doi:10.1093/femsle/fnag107)
Supplement: Supplementary file 1 [file fnag107_supplemental_files.zip › Supplementary Material.docx]

**Supplementary Material Description**

*This supplementary file presents the development process of the Community Microbiology Literacy Scale (CMLS), including the initial item pool, expert-based refinement stages, item-level content validity indices, and the mapping of retained items to the final validated version.*

**Supplementary Table S1.** Initial Item Pool of the Community Microbiology Literacy Scale (CMLS) (103 items)

| **Original Item Number** | **Initial Draft Item** | **I-CVI** |
| --- | --- | --- |
| **1** | I know how to define bacteria, fungi, and viruses and distinguish between them. | 0.750 |
| **2** | I know the optimal conditions required for bacterial growth. | 0.625 |
| **3** | I know the optimal conditions required for fungal growth. | 0.375 |
| **4** | I know the optimal conditions required for viral replication. | 0.750 |
| **5** | I know the differences between the cellular structures of bacteria, fungi, and viruses. | 0.750 |
| **6** | I know how to define prokaryotic and eukaryotic cells and distinguish between them. | 0.250 |
| **7** | I know the genetic differences between bacteria, fungi, and viruses. | 0.250 |
| **8** | I know the meaning of the term “Microbiology”. | **1.000** |
| **9** | Which of the following concepts is included within the term “microorganism”? | **1.000** |
| **10** | I know what microorganisms are. | **1.000** |
| **11** | I know what antibiotics, antifungals, and antivirals are and understand the differences between them. | 0.500 |
| **12** | I know the role of vaccines in protecting public health. | **1.000** |
| **13** | I am familiar with the concept of rational drug use. | 0.500 |
| **14** | I know the difference between sterilization and disinfection. | 0.500 |
| **15** | I know that viruses require a living host cell to replicate. | 0.750 |
| **16** | I know that enveloped viruses can be inactivated by soap, detergents, or antiseptics. | 0.625 |
| **17** | I know that non-enveloped viruses are generally less affected by soap, detergents, or antiseptics. | 0.625 |
| **18** | My knowledge and education in microbiology helped me manage my life during the pandemic period. | 0.750 |
| **19** | I know that antibiotics are not effective against viral infections such as influenza. | **1.000** |
| **20** | I know that bacteria can develop resistance to antibiotics over time. | 0.750 |
| **21** | I know that bacteria can develop resistance to different antibiotics. | 0.750 |
| **22** | I know that the term “hand sanitizer” should more accurately be referred to as “hand antiseptic”. | 0.375 |
| **23** | I know that vaccines provide protection by being administered before infection occurs. | **0.875** |
| **24** | I know that vaccines are safe and that adverse effects are rare. | 0.625 |
| **25** | I know the relative sizes of bacteria, viruses, and fungi and understand the differences between them. | **1.000** |
| **26** | I know the relative sizes of bacteria, viruses, and fungi and understand the differences between them. | 0.625 |
| **27** | I know which microorganisms can be filtered according to the pore size of masks. | 0.625 |
| **28** | I know the concept of mutation and why mutations are common in RNA viruses. | 0.500 |
| **29** | I am familiar with the concepts of isolation, quarantine, pandemic, social distancing, and hand hygiene. | 0.500 |
| **30** | I know that the use of cologne does not eliminate microorganisms. | 0.625 |
| **31** | I know the antimicrobial effect of ultraviolet (UV) radiation. | 0.500 |
| **32** | I know that microorganisms have been used as biological weapons. | **1.000** |
| **33** | I know that microorganisms can cause food spoilage and that consumption of contaminated food may result in food poisoning. | 0.750 |
| **34** | I am aware of the existence of the human microbiota. | 0.375 |
| **35** | I know that both beneficial and harmful microorganisms exist within the microbiota. | 0.375 |
| **36** | I know the microbial differences between cesarean section delivery and vaginal delivery. | 0.625 |
| **37** | I know the microbial differences between breast milk and infant formula. | 0.750 |
| **38** | I know the role of microorganisms in the production of foods such as pickles and bread. | 0.625 |
| **39** | I know that drinking water and recreational water (e.g., swimming pools) should be chlorinated to reduce microbial load. | 0.500 |
| **40** | I know that air hand dryers may carry a risk of microbial contamination after hand washing. | 0.625 |
| **41** | I know the role of microbial activity in the decomposition of environmental and biological waste. | 0.625 |
| **42** | I know that diseases such as measles, diphtheria, and poliomyelitis have been controlled through vaccination. | **1.000** |
| **43** | I know the benefits of probiotics and prebiotics for human health. | 0.500 |
| **44** | I know that microbiology studies organisms too small to be seen with the naked eye. | 0.375 |
| **45** | I know that microscopes are used to study microorganisms in microbiology. | **1.000** |
| **46** | My knowledge of microbiology helped me adopt preventive measures such as social distancing during the pandemic period. | 0.375 |
| **47** | I became aware of the importance of social distancing during the pandemic period. | 0.375 |
| **48** | I know what a microorganism is. | 0.625 |
| **49** | I know what a bacterium is. | 0.500 |
| **50** | I know what a virus is. | **1.000** |
| **51** | I know what a fungus is. | 0.625 |
| **52** | I know that fungi can cause disease in humans. | 0.625 |
| **53** | I know that bacteria can be beneficial as well as harmful. | 0.500 |
| **54** | I know that viruses can infect humans, animals, and plants. | 0.375 |
| **55** | I know that antibiotics are effective only against bacteria. | 0.500 |
| **56** | I know that antibiotics should not be used without medical advice. | 0.625 |
| **57** | I know that unnecessary antibiotic use contributes to antimicrobial resistance. | 0.625 |
| **58** | I know that incomplete antibiotic treatment may lead to resistance development. | **1.000** |
| **59** | I know that antibiotic resistance is an important public health problem. | **1.000** |
| **60** | I know that hand hygiene reduces the transmission of microorganisms. | **1.000** |
| **61** | I know that proper handwashing duration is important for preventing infection. | 0.625 |
| **62** | I know that cleaning and disinfection are not the same process. | 0.375 |
| **63** | I know that microorganisms can survive on surfaces for varying periods of time. | 0.500 |
| **64** | I know that vaccination protects both individuals and communities. | 0.500 |
| **65** | I know that vaccines stimulate the immune system to develop protection. | 0.625 |
| **66** | I know that vaccination contributes to herd immunity. | 0.625 |
| **67** | I know that vaccines undergo safety and effectiveness evaluations before approval. | **1.000** |
| **68** | I know that misinformation may negatively affect vaccination decisions. | 0.500 |
| **69** | I know that infectious diseases can spread rapidly within communities. | 0.375 |
| **70** | I know that early diagnosis and infection control practices reduce disease transmission. | 0.500 |
| **71** | I know that personal hygiene practices are important in preventing infectious diseases. | 0.500 |
| **72** | I know that some infectious diseases can be prevented through vaccination. | 0.500 |
| **73** | I know that not all microorganisms are harmful to human health. | **1.000** |
| **74** | I know that microorganisms play important roles in environmental balance. | 0.375 |
| **75** | I know that microorganisms contribute to ecosystem functioning and sustainability. | **1.000** |
| **76** | I know that environmental conditions may influence the spread of microorganisms. | **1.000** |
| **77** | I know that microorganisms can adapt to changing environmental conditions. | **1.000** |
| **78** | I know that climate change may affect the distribution of infectious diseases. | **1.000** |
| **79** | I know that food hygiene practices reduce microbial contamination. | **1.000** |
| **80** | I know that proper storage conditions help prevent microbial growth in foods. | 0.500 |
| **81** | I know that safe drinking water is important for preventing infectious diseases. | 0.625 |
| **82** | I know that microorganisms may spread through contaminated food and water. | 0.625 |
| **83** | I know that microorganisms may spread through respiratory droplets. | 0.375 |
| **84** | I know that microorganisms may spread through direct or indirect contact. | 0.375 |
| **85** | I know that some microorganisms can remain viable outside the human body for a period of time. | 0.750 |
| **86** | I know that infection prevention measures reduce disease transmission. | 0.625 |
| **87** | I know that antimicrobial resistance may limit treatment options for infectious diseases. | 0.375 |
| **88** | I know that responsible antimicrobial use contributes to reducing resistance development. | 0.375 |
| **89** | I know that healthcare-associated infections are an important public health concern. | 0.500 |
| **90** | I know that microorganisms may develop resistance to antimicrobial agents over time. | 0.625 |
| **91** | I know that public awareness plays an important role in infection prevention and control. | 0.625 |
| **92** | I know that scientific information is important for making health-related decisions. | **1.000** |
| **93** | I know that misinformation may negatively influence public health behaviors. | 0.500 |
| **94** | I know that community awareness contributes to controlling infectious disease outbreaks. | 0.500 |
| **95** | I know that microbiology knowledge may contribute to healthier daily-life practices. | 0.500 |
| **96** | I know that microbiology education may improve public health awareness. | 0.625 |
| **97** | I know that understanding microorganisms contributes to informed health decisions. | **1.000** |
| **98** | I know that microbiology literacy may influence preventive health behaviors. | 0.375 |
| **99** | I know that improving microbiology literacy may contribute to public health. | 0.500 |
| **100** | I know that microbiology is relevant to daily life and community health. | 0.500 |
| **101** | I know that microorganisms may affect human health in both beneficial and harmful ways. | **1.000** |
| **102** | I know that microorganisms play important roles in maintaining ecological balance. | **1.000** |
| **103** | I am familiar with the names and characteristics of different groups of microorganisms. | 0.375 |

***Note.*** This table presents the initial 103-item pool generated during the development of the Community Microbiology Literacy Scale (CMLS). Items were created based on literature review and theoretical conceptualization of microbiology literacy and were subsequently subjected to expert evaluation, content validity assessment, and psychometric refinement to obtain the final validated version.

**Supplementary Table S2.** Content Validity Index (CVI) Results of the Retained Items

| **Final Item Number** | **Original Item Number** | **I-CVI** |
| --- | --- | --- |
| Item 1 | 8 | 1.000 |
| Item 2 | 9 | 1.000 |
| Item 3 | 10 | 1.000 |
| Item 4 | 12 | 1.000 |
| Item 5 | 19 | 1.000 |
| Item 6 | 23 | 0.875 |
| Item 7 | 24 | 1.000 |
| Item 8 | 32 | 1.000 |
| Item 9 | 42 | 1.000 |
| Item 10 | 45 | 1.000 |
| Item 11 | 50 | 1.000 |
| Item 12 | 58 | 1.000 |
| Item 13 | 59 | 1.000 |
| Item 14 | 60 | 1.000 |
| Item 15 | 67 | 1.000 |
| Item 16 | 73 | 1.000 |
| Item 17 | 75 | 1.000 |
| Item 18 | 76 | 1.000 |
| Item 19 | 77 | 1.000 |
| Item 20 | 78 | 1.000 |
| Item 21 | 79 | 1.000 |
| Item 22 | 92 | 1.000 |
| Item 23 | 97 | 1.000 |
| Item 24 | 101 | 1.000 |
| Item 25 | 102 | 1.000 |
| **S-CVI/Ave** | | **0.995** |

Item numbering corresponds to the original draft version and the final retained version after expert review.

**Supplementary Table S3.** Community Microbiology Literacy Scale (in Turkish)

| **Toplumun Mikrobiyoloji Okur Yazarlık Ölçeği** | | | | | | |
| --- | --- | --- | --- | --- | --- | --- |
|  |  | **Kesinlikle Katılmıyorum** | **Katılmıyorum** | **Ne Katılıyorum Ne Katılmıyorum** | **Katılıyorum** | **Kesinlikle Katılıyorum** |
|  | **Temel Mikrobiyoloji Bilgisi** | | | | | |
| **1** | **TMB_1.** Mikroorganizmaların neler olduğunu biliyorum. |  |  |  |  |  |
| **2** | **TMB _2.** Mikroorganizmaların hem zararlı hem de yararlı türleri olduğunu biliyorum. |  |  |  |  |  |
| **3** | **TMB_3.** Mikroorganizmaların insan sağlığını nasıl etkilediğini biliyorum. |  |  |  |  |  |
| **4** | **TMB_4.** Mikroorganizmaların nasıl bulaştığını biliyorum (örneğin hava, su veya temas yoluyla). |  |  |  |  |  |
| **5** | **TMB _5.** Mikroorganizmaların gıdaların bozulmasına neden olabileceğini ve kontamine (mikroorganizma bulaşmış) gıdaların tüketilmesinin insanlarda gıda zehirlenmesine yol açabileceğini biliyorum. |  |  |  |  |  |
| **6** | **TMB_6.** Bakteriler ile virüsler arasındaki farkları biliyorum. |  |  |  |  |  |
| **7** | **TMB_7.** Bakteriler ile mantarlar arasındaki farkları biliyorum. |  |  |  |  |  |
| **8** | **TMB_8.** Küf ve mayaların mantar türleri olduğunu biliyorum. |  |  |  |  |  |
| **Enfeksiyon Kontrolü Farkındalığı** | | | | | | |
| **9** | **EKF_1.** Mikroorganizmaların neden olduğu enfeksiyon hastalıklarını önlemede el hijyeninin önemini biliyorum. |  |  |  |  |  |
| **10** | **EKF_2.** Enfeksiyon hastalıklarının yayılmasını önlemede sosyal mesafe ve maske kullanımının önemini biliyorum. |  |  |  |  |  |
| **11** | **EKF_3.** Mikroorganizmaların bulaşmasını önlemek için günlük hijyen alışkanlıklarımı değiştirdiğime inanıyorum. |  |  |  |  |  |
| **12** | **EKF_4.** Toplumda yaygın olan hijyen hatalarının enfeksiyon riskini artırdığını biliyorum. |  |  |  |  |  |
| **13** | **EKF_5.** Mikrobiyoloji bilgimin hijyen uygulamalarımı geliştirdiğine inanıyorum. |  |  |  |  |  |
| **14** | **EKF_6.** Mikrobiyoloji bilgim sayesinde pandemi sürecinde sosyal mesafe gibi koruyucu önlemler aldım. |  |  |  |  |  |
| **15** | **EKF_7.** Günlük yaşamda hijyenik olmayan alışkanlıkların mikrobiyal bulaşma riskini artırdığını biliyorum. |  |  |  |  |  |
| **Antibiyotik ve Direnç Farkındalığı** | | | | | | |
| **16** | **ADF_1.** Antibiyotiklerin yalnızca bakteriyel enfeksiyonlara karşı etkili olan kimyasal ilaçlar olduğunu biliyorum. |  |  |  |  |  |
| **17** | **ADF_2.** Antibiyotiklerin grip gibi viral enfeksiyonlarda etkili olmadığını biliyorum. |  |  |  |  |  |
| **18** | **ADF_3.** Gereksiz antibiyotik kullanımının bakterilerin antibiyotiklere karşı direnç geliştirmesine yol açtığını biliyorum. |  |  |  |  |  |
| **19** | **ADF_4.** Antibiyotik direncinin küresel halk sağlığını olumsuz etkilediğini biliyorum. |  |  |  |  |  |
| **Aşı Farkındalığı** | |  |  |  |  |  |
| **20** | **AF_1.** Aşıların halk sağlığını korumada önemli bir rol oynadığını biliyorum. |  |  |  |  |  |
| **21** | **AF_2.** Aşıların, kişi hastalanmadan önce uygulandığında koruyuculuk sağladığını biliyorum. |  |  |  |  |  |
| **22** | **AF_3.** Aşıların güvenli olduğunu ve ciddi yan etkilerin nadir görüldüğünü biliyorum. |  |  |  |  |  |

**Figure S1.** Development of the Microbiology Literacy Scale: Item Selection and Content Validation
